# A concise overview of advancements in ultrasensitive biosensor development

**DOI:** 10.3389/fbioe.2023.1288049

**Published:** 2023-11-28

**Authors:** Ammara Shahid, Fazila Nazir, Muhammad Jawad Khan, Sana Sabahat, Aisha Naeem

**Affiliations:** ^1^ Department of Chemistry, COMSATS University Islamabad, Islamabad, Pakistan; ^2^ Department of Biosciences, COMSATS University Islamabad, Islamabad, Pakistan; ^3^ QU Health, Qatar University, Doha, Qatar

**Keywords:** electrochemical sensor, nanoparticles, miRNA, limit of detection, biosensor

## Abstract

Electrochemical biosensing has evolved as a diverse and potent method for detecting and analyzing biological entities ranging from tiny molecules to large macromolecules. Electrochemical biosensors are a desirable option in a variety of industries, including healthcare, environmental monitoring, and food safety, due to significant advancements in sensitivity, selectivity, and portability brought about by the integration of electrochemical techniques with nanomaterials, bio-recognition components, and microfluidics. In this review, we discussed the realm of electrochemical sensors, investigating and contrasting the diverse strategies that have been harnessed to push the boundaries of the limit of detection and achieve miniaturization. Furthermore, we assessed distinct electrochemical sensing methods employed in detection such as potentiometers, amperometers, conductometers, colorimeters, transistors, and electrical impedance spectroscopy to gauge their performance in various contexts. This article offers a panoramic view of strategies aimed at augmenting the limit of detection (LOD) of electrochemical sensors. The role of nanomaterials in shaping the capabilities of these sensors is examined in detail, accompanied by insights into the chemical modifications that enhance their functionality. Furthermore, our work not only offers a comprehensive strategic framework but also delineates the advanced methodologies employed in the development of electrochemical biosensors. This equips researchers with the knowledge required to develop more accurate and efficient detection technologies.

## Introduction

Biosensing, or the detection and analysis of biological entities, is important in many domains of science and technology such as medicine, environmental monitoring, and food safety. The capacity to identify and measure biological molecules and organisms precisely and quickly is critical for disease diagnosis, environmental evaluation, and maintaining the safety and quality of food items (Singh et al., 2021). In recent years, electrochemical biosensing has emerged as a powerful tool in this field, offering a number of advantages over conventional detection (Singh et al., 2021). These biosensors convert a target analyte recognition event into an electrical signal using electrochemical principles, allowing for sensitive and targeted detection. To deliver accurate and trustworthy findings, they incorporate transducers, bio-recognition components, and electrochemical processes. Electrochemical biosensors now offer significantly better sensitivity, selectivity, mobility, and cost-effectiveness owing to advancements in nanotechnology, biochemistry, and microfabrication (Luong et al., 2020).

One of the main benefits of electrochemical biosensors is their ability to do real-time measurements with high sensitivity, specificity, and resolution (Sumitha and Xavier, 2023). Amperometric biosensors, for example, measure the electrical current produced as a result of an analyte’s redox interaction with an electrode surface and provide precise and quantitative information about the concentration of the target molecule (Sumitha and Xavier, 2023). Potentiometric biosensors detect the potential difference between two electrodes in a solution, whereas impedance-based biosensors analyze changes in the system’s electrical impedance (Banakar et al., 2022). In addition, the incorporation of nanoparticles has significantly improved the performance of electrochemical biosensors. Many nanomaterials such as carbon-based, metallic, quantum dots, and nanowires have special properties such as a larger surface area, higher catalytic activity, and superior electron transfer kinetics that contribute significantly to amplified signals and increased sensor performance (Pérez-Fernández and de la Escosura-Muniz, 2022).

Electrochemical biosensors can be used to detect various biological molecules such as enzymes, antibodies, nucleic acids, aptamers, and molecularly imprinted polymers. An electrode is employed as a solid support for immobilization of these biomolecules depending on the specificity of chemical groups attached to the surface of electrodes which is necessary for the effective detection of the complementary target molecule (Naresh and Lee, 2021). Their coupling with electrochemical transducers facilitates the translation of biological interactions into detectable electrical impulses. Biological interactions can be transformed into measurable electrical impulses by integration with the electrochemical transducers (Lu et al., 2014; Banakar et al., 2022).

Electrochemical biosensors are appropriate for point-of-care testing and field applications because these technologies allow for reduced sample and reagent amounts, shorter analysis times, and increased automation (Goda et al., 2023). Microfluidics and lab-on-a-chip technologies have transformed the area of electrochemical biosensing by enabling sample preparation and analysis to be miniaturized, portable, and integrated. These biosensors have been demonstrated to be crucial in clinical diagnostics and on-the-spot testing for identifying infectious microorganisms, detecting disease biomarkers, and monitoring therapeutic prescription levels (Valera et al., 2023). Environmental monitoring uses electrochemical biosensors to find contaminants, heavy metals, and pathogens in water, soil, and air (He et al., 2023). Despite all these developments, further research is inevitable in miniaturization, the creation of reliable and precise sample preparation procedures, and the incorporation of data processing algorithms (Mehrvar and Abdi, 2004; Kucherenko et al., 2020; Mariani et al., 2022).

In this comprehensive review article, we explored diverse fabrication strategies involving various nanocomposites to provide a deeper insight into the electrochemical detection phenomena of ultrasensitive biosensors. Throughout the review, we meticulously emphasize and draw comparisons among different strategies that were aimed at two key objectives: augmenting the limit of detection (LOD) and advancing the miniaturization process of electrochemical sensors. We shed light on the intricacies of each approach, offering insights into their effectiveness and potential applications. Our focus extends to a detailed examination of the various nanomaterials currently harnessed within electrochemical sensors, where we illuminate their respective merits and demerits. We specifically pinpointed most recently reported the top ten strategies to develop ultrasensitive biosensors. This evaluation is further refined by comparing the performance of distinct sensors or sensing methods including potentiometers, amperometers, conductometers, colorimeters, transistors, and electrical impedance spectroscopy, which are employed for the purpose of detection. The introduction of nanomaterials can improve electrochemical sensors in several aspects, such as sensitivity, selectivity, response times, detection limits, and detection range. Along with working in a variety of electrochemical ways, these sensors are also portable and energy efficient. *In-situ* monitoring is made possible by nanomaterials, which are also used extensively in the environmental and medical monitoring fields. In a nutshell, this article offers a succinct overview of high-performance biosensor development, focusing on nanomaterial utilization, electrochemical sensing, and fabrication strategies.

### Diversity of nanomaterials in electrochemical sensors

The utilization of nanomaterials capitalizes on their large surface area-to-volume ratio, providing additional binding sites for bio-recognition and element immobilization, consequently augmenting the potential for target analyte binding, and simultaneously contributing significantly to the miniaturization process. Nanomaterials play a pivotal role in boosting signal transduction and enhancing detection limits, acting as effective amplifiers within the electrochemical sensors (Bezinge et al., 2020). Microscale environment within sensor technology addresses many challenges such as non-uniform pH distribution, electrical distortion, and uneven application of electrical perturbation (Algamili et al., 2021). These problems are managed primarily through the control of spatiotemporal fluctuations and the choice of nanomaterials (Ferrag and Kerman, 2020). Micro/nanopatterning for biosensor design, microfluidic biosensors, and microelectromechanical Systems (MEMs)-based biosensors exemplify microfabricated sensing devices (Algamili et al., 2021).

**FIGURE 1:**
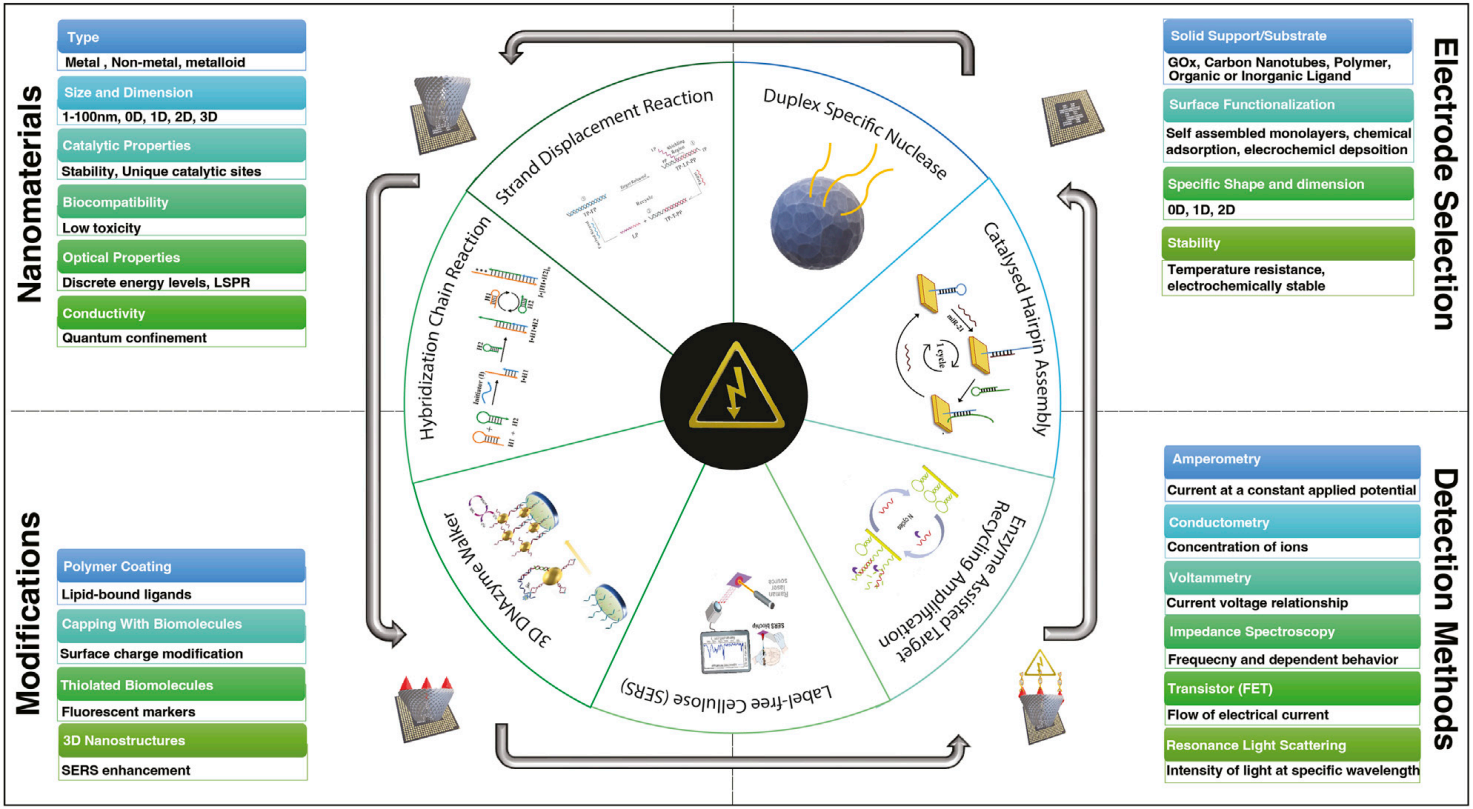
Diversity of nanomaterials, electrode selection, ligand variability, and electrochemical sensing techniques in the framework of biosensor development for detecting various miRNAs.

The development and fabrication of electrochemical sensors largely revolve around incorporating various types of nanoparticles onto electrode surfaces (Figure 1; Table 1). These nanoparticles encompass metallic elements such as gold (Au), silver (Ag), cadmium (Cd), ruthenium (Ru), terbium (Tb), molybdenum (Mo), platinum (Pt), copper (Cu), palladium (Pd), cobalt (Co), indium (In), osmium (Os), and lead (Pb), as well as non-metallic elements like carbon (C), silicon (Si), and phosphorus (P). Additionally, substrates derived from organic sources, such as metal-organic frameworks (MOFs) (Tian et al., 2019; Liu et al., 2022), and polyaniline (PAn) (Fan et al., 2007) have also been employed in research (Figure 1; Table 1). The choice of nanomaterial depends on the specific requirements of the biosensing application. The article explores the integration of various nanomaterials into electrochemical sensors, outlining the merits and drawbacks associated with their usage in sensor enhancement along with the integral role they play in the miniaturization of biosensors.

**TABLE 1:** The electrochemical detection methods with their linear range and limit of detection for various miRNAs.

Nanomaterials used	Electro-chemical method	Detection limit (fM)	Linear range (fM)	Target analyte	Reference
Silicon Nanowire	AMP	1	NA	miR-21	Liu et al. (2012)
AuNP	AMP	0.044, 0.0136	NA	RSV DNA, let-7a	Li et al. (2023)
Ruthenium oxide NP-catalyzed polyaniline	AMP	2	NA	Let-7c	Kim and Kang (2023)
AuNP	CV	0.12	2.5–2.5 × 10^7^	miR-21	Liu et al. (2022)
Pyrrolidinyl peptide nucleic acid/Ag NF- GCE	CV	0.20	NA	miR-21	Ranjan Srivastava et al. (2022)
Cobalt Ferrite Magnetic NP	CV	0.3	1–2 × 10^6^	miR-21	Wang et al. (2015b)
Platinum@Cerium oxide NS	CV/EIS	1.41	10–1 × 10^6^	miR-21	Mohammadnejad et al. (2023)
AuNP	DPV	0.12	10–2 × 10^3^	miR-182	Yoon et al. (2022)
AuNP	DPV	0.058	1–2 × 10^3^	miR-182	Kim et al. (2018)
Magnetic NP (DNA1/Fe3O4 NPs/Thi and DNA2/Fe3O4 NPs/Fc)	DPV	0.28, 0.36	NA	miR-141, -21	Szunerits et al. (2022)
Catalytic hairpin assembly + B12	DPV	4.5	10–1 × 10^7^	miR-141	Khazaei et al. (2023)
Catalytic hairpin assembly	DPV	3.608	10–1 × 10^6^	miR-21	Das et al. (2022)
AuNP@Mxenes	DPV	0.204, 0.138	0.5–5 × 10^7^	miR-21, -141	Li et al. (2021)
AuNP/polypyrrole-reduced graphene oxide	DPV	1.57	10–5 × 10^6^	miR-16	Gao et al. (2020)
Iron oxide/Cerium oxide/Au	DPV	0.33	1–1 × 10^6^	miR-21	Tian et al. (2023)
PdNP	DPV	0.0086	0.05–1 × 10^2^	miR-21	Tran et al. (2013)
Iron-embedded nitrogen-rich carbon NT	DPV	0.853	1–1 × 10^6^	miR-486	Bao et al. (2019)
Carbon Spheres-Molybdenum disulphide	DPV	0.016	0.1–1 × 10^5^	miR-21	Wang et al. (2021b)
T7 exonuclease/Copper NP	DPV	0.045	1–1 × 10^3^	miR-141	Deng et al. (2018)
AgNPs@N,O-C BLHS	DPV	0.01	NA	ctDNA	Cui et al. (2019)
DNA hairpin probes (cDNA, H1, and H2)	EIS	4.63	10–5 × 10^4^	miR-21	Chen et al. (2022)
Graphene oxide@AuPd NP	ECL	0.0319	0.1–1 × 10^6^	miR-141	Safari et al. (2023)
AuPd alloy seeds NP/Graphitic carbon nitride NS	ECL	0.331	1–1 × 10^7^	miR-141	Naikoo et al. (2021)
Au@luminol NPs	ECL	0.004	0.01–1 × 10^3^	miR-21	Xu et al. (2020)
SnO2 QDs	ECL	0.002	0.01–1 × 10^5^	miR-21	Meng et al. (2020)
ABEI@AuPd NPs	ECL	0.0319	0.1–1 × 10^8^	miR-141	Safari et al. (2023)
Poly (9,9-di-n-octylfluorenyl-2,7-diyl) polymer NPs	ECL	0.017	0.05–1 × 10^5^	miR-155	Castro et al. (2023)
DPA@Pe MCs	ECL	0.00414	0.01–1 × 10^3^	miR-21	Kim et al. (2020)
BP-CdTe QDs	ECL	0.029	NA	miR-126	Várallyay et al. (2008)
PtNPs-modified GCE	ECL	0.027	0.1–1 × 10^5^	HIV DNA	Fan et al. (2007)
Silicon nanowires	FET	1	N/A	Let-7b	Molla and Youk (2023)
Graphene oxide	FL	0.17	1 × 10^5^	miR-16,-21;-26a	Lao et al. (2006)
AgNC	FL	0.002	NA	miR-141	Gao and Yu (2007b)
Zirconium porphyrin MOF	FL	0.011	NA	miR-21	Chen et al. (2018)
Methylammonium lead halide QDs	PEC	0.005	0.01–2 × 10^7^	miR-155	Qin et al. (2023)
AuNP	SWV	0.03113	0.1–1 × 10^6^	miR-182–5p	Tian et al. (2019)
DNA circle capture probe @ tetrahedron DNA nanostructure	SWV	0.0189 0.0396	0.1–1 × 10^7^	miR-21,-155	Liu et al. (2018)
Pt@Copper MOFs	SWV	0.1	1–1 × 10^6^	miR-21,-141	Wang et al. (2021a)
Iron Oxide@AuNS	SWV	1.5, 1.8	5–2 × 10^6^	miR-21,-155	Sabahat et al. (2023)
DNA tetrahedron nanostructures	SWV	0.01217	0.05–1 × 10^4^	miR-133a	Munusami et al. (2022)
Copper-based MOF @PtNP	SWV	0.3	0.5–1 × 10^5^	miR-155	Walcarius et al. (2013)
AuNPs-P-DM probe	SWV	0.0331	0.1–1 × 10^6^	miR-21	Wang et al. (2020b)

Au, gold; NP, nanoparticles; Pd, palladium; NS, nanospheres; NT, nanotubes; DM, DNAzyme; P, protected strand; MOF, metal organic framework; NC, nanoclusters; Ag, silver; QDs, quantum dots; Pt, platinum; SnO_2_, tin oxide; GCE, glassy carbon electrode; BP, black phosphorus; DPA, 9,10-diphenylanthracene; Pe, perylene; MCs, microcrystals; ABEI, N-(4-Aminobutyl)-N-(elthylisoluminol); BLHS, broom-like hierarchical nanomaterials; NT, nanotubes; NF, nanoflower; Fc, ferrocene; AMP, amperometry; DPV, differential pulse voltammetry; SWV, square wave voltammetry; CV, cyclic voltammetry; EIS, electrochemical impedance spectroscopy; ECL, electrochemiluminescence; FL, fluorescence; FET, field-effect transistor; PEC, photoelectrochemical.

### Non-metallic and metalloid nanomaterials

With the distinctive properties they possess, carbon-based nanomaterials like carbon nanotubes (CNTs) and graphene have attracted a lot of interest in the field of electrochemical biosensing. CNTs along with graphene can be utilized as transducers or to modify electrodes (Deng et al., 2018). Their combination improves the kinetics of electron transfer and improves the immobilization of bio-recognition components due to their high surface area and good conductivity, which offers a reliable analyte detection platform (Naresh and Lee, 2021). Also, by functionalizing carbon-based nanomaterials with specific groups such as Fe_2_O_3_, Mg(OH)_2_, graphene oxides, and polymers for selective binding, improving the sensitivity and selectivity of biosensors significantly (Tran et al., 2013; Chen et al., 2018; Deng et al., 2018; Wang et al., 2021a).

Using an electroactive polymer and interconnected network of CNTs, an—unlabelled and reagent-free sensor design was introduced in 2013 (Table 1) (Liu et al., 2021). The polyfluorene contained in the polymer backbone exhibits high fluorescence quantum yield, photo-stability, as well as non-toxic and easy structural modification, which gives the nanostructured polymer film a highly distinct electroactivity in the cathodic potential domain in a neutral aqueous medium which response strongly to miRNA responses due to higher polymer electroactivity (Liu et al., 2021). Similarly, in research thionin loading capacity was studied on shorter multi-walled carbon nanotubes (S-MWCNTs) and modified multi-walled carbon nanotubes (A-MWCNTs) (Deng et al., 2018). Because of the enormous effective surface area of MWCNTs, quick electron shuttle of MWCNTs, and high-loaded thionin on S-MWCNTs (Huang et al., 2020) developed a uniform, large-area, layered graphene composite of graphene oxide/graphene (GO/G).

Furthermore, a fluorescent-based sensing framework was established using the integration of carbon-based nanomaterials, and the process was initiated through target recycling activated by duplex-specific nuclease (DSN). Because of the weak contact between the short DNA segments and GO, GO induces a high fluorescence emission (Guo et al., 2014). Another study (Chen et al., 2018), introduced a novel sensing substrate involving the assembly of carbon spheres coated with molybdenum disulfide nanosheets (CS-MoS_2_ NSs). The combination of CS-MoS_2_ into a sensor configuration contributed to a high specific surface area, improved stability, and enhanced dispersibility of the sensor. The researchers focused on a target recycling amplification technique known as Catalytic hairpin assembly (CHA) to deal with the DNA structure transition which hinders the access of quenching probes due to steric hindrance (Jiang et al., 2020; Zhang et al., 2020).

Further advancements are also carried out based on non-metallic nanomaterials have gained popularity due to their low cost, ease of manufacture, biocompatibility, and considerable electrochemical and optical capabilities. Graphene and its derivatives, CNTs, and carbon dots have been explored in the literature for the development of various electrochemical and optical cancer-detecting biosensors (Wang et al., 2021b; Castro et al., 2023; Safari et al., 2023).

Non-metallic nanoparticle-based biosensors are proven to be instrumental in establishing a microscale environment. The endeavor to downsize non-metallic nanoparticle-based biosensors achieved a significant advancement (Sobhanie et al., 2022; Castro et al., 2023; Khazaei et al., 2023). The methodology entailed combining paper-based microfluidics with an electrochemical sensor, resulting in a feasible and efficient framework for creating small, cost-effective analytical devices (Das et al., 2022). Leveraging the unique properties of paper as a substrate, microchannels were formed to facilitate fluid transportation and manipulation within the context of paper-based microfluidics. The combination of these microfluidic capabilities with electrochemical sensing technologies leads to innovative approaches for the development of diminutive, efficient, and disposable sensing devices. These innovations have immense potential to revolutionize the landscape of diagnostic tools, offering an affordable and portable analytical solution across various biosensing applications.

In contrast to metallic sensors, sensors based on non-metallic and metalloid elements exhibit reduced sensitivity, selectivity, and range due to their lower abundance of free electrons essential for detection. Furthermore, their increased vulnerability to environmental oxidation and degradation renders them less robust and enduring than their metallic biosensor counterparts (Kim et al., 2020; Naikoo et al., 2021).

### Metal nanoparticles

Metal nanoparticles like gold and silver nanoparticles are the most frequently used in the construction and design of electrochemical biosensors (Ndolomingo et al., 2020). These nanoparticles have special optical, electrical, and catalytic abilities that are used to improve the performance of biosensors (Mehmood et al., 2015; Ndolomingo et al., 2020). Due to their localized surface plasmon resonance (LSPR) properties, they can serve as signal amplifiers (Wang et al., 2015a). The analyte interacts with the bio-recognition component on the nanoparticle surface, changing the LSPR and resulting in detectable signals (Kangkamano et al., 2018). Metal nanoparticles not only function as redox catalysts but also provide increased surface area for immobilizing bio-recognition components, enhancing the sensitivity of biosensors (Gao and Yu, 2007a; Wang et al., 2015a; Hao et al., 2017).

The use of AuNP in conjunction with magnetic microbeads (MMBs) in the fabrication of DNA nanomachines amplified strand displacement reaction (SDR) signal, resulting in increased sensitivity and selectivity in electrochemical miRNA detection. The combination of AuNP-SA MMBs with 3D DNA nanomachines (DNM) utilizing a toehold-mediated SDR (TSDR) maintained a stable signal for AuNP-streptavidin MMBs, thus mitigating the influence of environmental factors (Lu et al., 2020). In contrast, a similar approach was used by replacing streptavidin with Fe_2_O_3_ (Gao et al., 2022), resulting in significantly improved detection sensitivity by using electrochemical detection as compared to the conventional method such as northern blotting (Várallyay et al., 2008) and reverse-transcription polymerase chain reaction (Lao et al., 2006). In another approach, 3D DNAzyme walker and the gold nanoparticles/graphene aerogels carbon fiber paper-based (AuNPs/GAs/CFP) combined with streptavidin-modified magnetic beads (MBs) were used to detect miR-155 (Zhao et al., 2023).

The field of biosensor miniaturization with metallic nanoparticles has witnessed several intriguing advancements. For early diagnosis of SAH-induced cerebral vasospasm and hydrocephalus, a team of researchers designed a label-free cellulose (SERS) biosensor chip with pH-functionalized, AuNP-enhanced LSPR effects (Kim et al., 2018; Ranjan Srivastava et al., 2022; Chandra et al., 2023; Ray et al., 2023). The label free cellulose SERS biosensor chip was integrated by transferring positively charged AuNPs onto a negatively charged cellulose substrate via a synthesis procedure. The zeta potential, nanostructural characteristics, nanocrystallinity, and computational calculation-based electric field distributions of cellulose-derived AuNPs were optimized and characterized to maximize LSPR phenomena. The miniaturization process facilitated high resolution, high sensitivity, and multiplexing of bioanalytics characterized to maximize the detection (Kim et al., 2018; Yoon et al., 2022). Another study (Ulucan-Karnak et al., 2023), designed a sensor based on metal-oxide nanomaterials (MONs) which played a substantial role in the development of flexible/wearable sensors due to their tunable band gap, low-cost, wide specific area, ease of fabrication, and multiplexing properties.

While metallic nanoparticles possess numerous adjustable characteristics, their potential cytotoxicity to living tissues and cells poses limitations on their biosensor applications. Drastic variations in pH or temperature can render metallic nanoparticles unstable, compromising sensitivity and selectivity and potentially leading to false positive or negative results. Maintaining consistent production of metallic nanoparticles is challenging, leading to performance discrepancies among biosensors. Furthermore, metallic biosensors exhibit a narrower detection range in comparison to optical biosensors (Cho et al., 2020; Naresh and Lee, 2021).

### Nanowires and quantum dots

Nanowires (NW), quantum dots (QDs), metal nanoparticles, and carbon-based nanomaterials all have special features that may be customized to certain biosensing uses. Given their distinctive optical and electrical properties, NW and QDs are appealing materials for electrochemical biosensors (Ozkan-Ariksoysal and Uslu, 2021) (Table 1). QDs are semiconductor nanocrystals with remarkable detection-grade photoluminescence signals and size-dependent fluorescence characteristics. They are functionalized with bio-recognition elements and can be utilized as labels for target analyte detection to enable multiplexed analysis (Algar et al., 2010; Yuan et al., 2017). They facilitate improvements in healthcare, environmental monitoring, and other sectors by improving the performance of electrochemical biosensors.

The uses and advantages of QDs in electrochemical sensing have been extensively documented recently (He et al., 2023; Pourmadadi et al., 2023). The incorporation of CH3NH3PbI3 QDs was documented to lead to a notable improvement in the sensitivity and light-absorption capabilities of ZnO-NSs (Pang et al., 2016). These CH3NH3PbI3 QDs, characterized by their optimal band gap energy and efficient sunlight absorption, offer a novel approach for enhancing the sensitivity of ZnO-NSs and have been seamlessly integrated into a photoelectrochemical (PEC) aptasensor for miRNA detection (Pang et al., 2016). However, the utilization of QDs comes with some drawbacks as well, particularly their toxicity. Future research is warranted toward employing less toxic QDs, such as graphene-based QDs with unique optical properties, which hold promise for diverse applications including bioimaging and biosensing (Mohamed et al., 2021).

Nanowires, on the other hand, have a high aspect ratio that enables direct electron transmission from the analyte to the electrode surface (Zhou et al., 2023). Their one-dimensional structure makes it easier to immobilize bio-recognition components and increases the efficiency of charge transfer, increasing the sensitivity of the biosensors (Gao et al., 2013). A study (He et al., 2017) explored the potential of silicon nanowire (SiNW) biosensors which are a promising tool for miRNA detection due to their rapid reaction times and heightened sensitivity. They present a well-established method involving poly-silicon nanowire biosensors for detecting miRNA (let-7b), achieving LOD of 1 fM (femtomolar) (He et al., 2017) (Table 1). In another study, PAn-modified SiNW was used to detect miRNAs by means of a nano-gapped microelectrode-based biosensor. The conductivity of the deposited PAn NW is directly proportional to the amount of hybridized miRNA. Under optimal conditions, this approach enables good detection of target miRNA with a LOD of 5.0 fM (Fan et al., 2007). Recent developments have been made on NW sensors by incorporating several nanoparticles with specific binding ability (Tran et al., 2023; Zhou et al., 2023).

Although these investigations yield remarkable detection limits, showcasing the potential of QDs and NW-based sensors, there remains a significant need for further investigation to tackle their constraints. These limitations encompass potential toxicity concerns and challenges associated with precise control over size, shape, and composition during the synthesis process (Zhou et al., 2023).

### Strategies for enhancing LOD of electrochemical sensors

**FIGURE 2:**
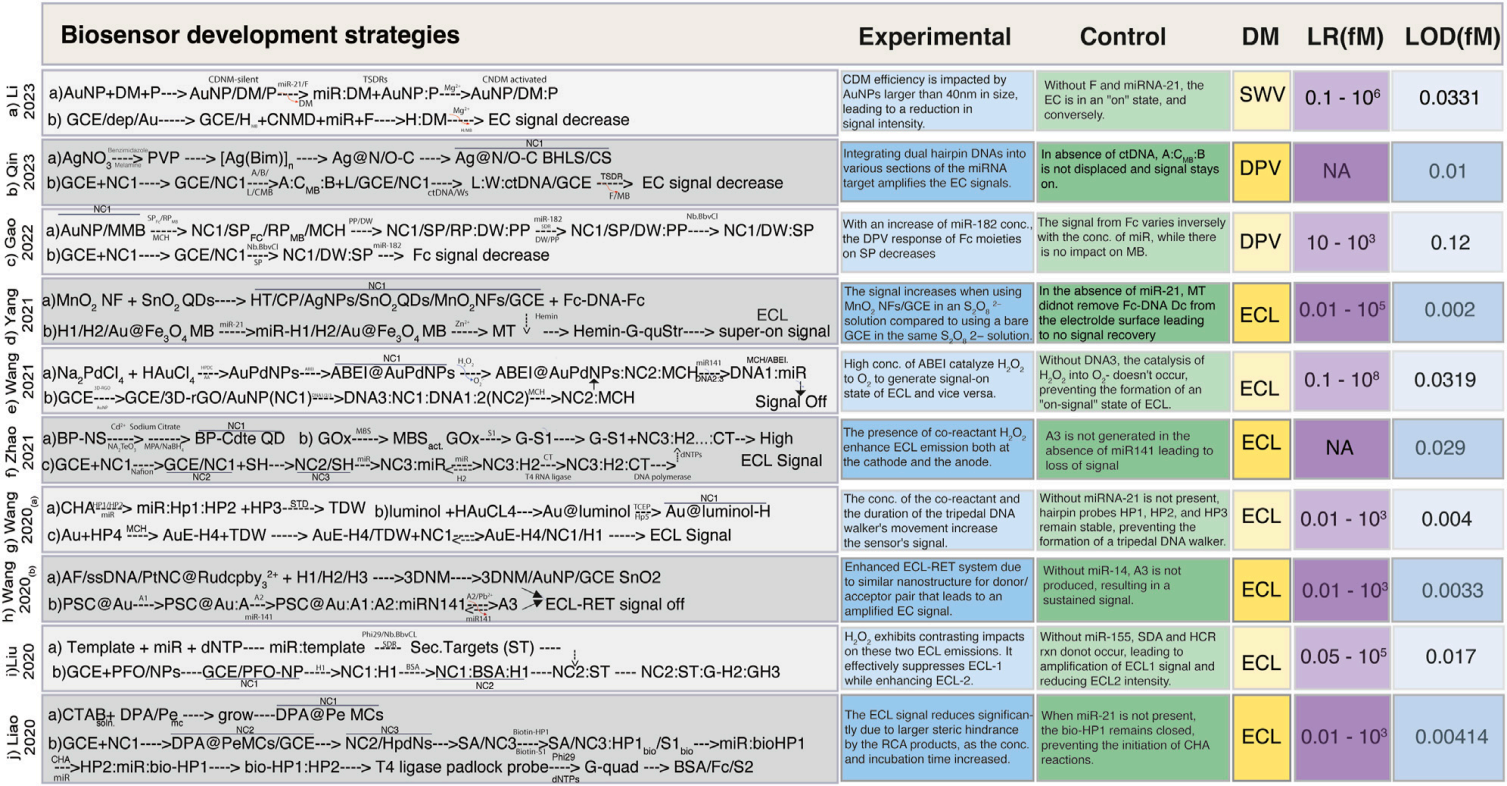
Simplified schemes of the fabrication of ultrasensitive biosensors. **(A)** Schematic illustration of the preparation of the CDNM via target-triggered TSDRs and the walking process of the CDNM in the presence of target miRNA (Li et al., 2023). **(B)** Schematic of Ag@N,O-C BLHS synthesis, and electrochemical sensing mechanism for ctDNA detection sensitized with Ag@N, O-C BLHS driven by DNA walker (Qin et al., 2023). **(C)** Construction of a ratiometric electrochemical sensor based on the 3D-DNA nanomachine with multiple hybridization and cleavage cycles for miRNA detection (Gao et al., 2022). **(D)** Fabrication scheme for the Ag NPs/SnO2 QDs/MnO2 NFs based-ECL biosensor for miR-21 detection (Yang et al., 2021a). **(E)** Creation of ABEI@AuPd NPs sensor with DNA nanomachines walking freely on ECL electrodes for the detection of miR-141 (Wang et al., 2021a). **(F)** BP-CdTe QDs biosensor construction and GOx conjugation to S1 for miR-126 detection (Zhao et al., 2021). **(G)** Schematic based on the CHA-tripedal DNA walker strategy along with the walking process of the tripedal DNA walker on the electrode of ECL for the detection of miRNA-21 (Wang et al., 2020a). **(H)** AF-PtNPs@Ru (dcbpy)^2/3+^ assembly with 3D DNM using target recycling amplification technology and the multiple ECL-RET biosensor for the detection of miR-141 (Wang et al., 2020b). **(I)** Schematic illustration of the preparation of ST, its assembly steps, and signal conversion mechanism of the ratiometric biosensor for detecting miR-155 (Liu et al., 2021). **(J)** DPA@Pe biosensor fabrication strategy based on affinity switch using CHA and RCA amplification strategy for miR-21 detection (Liao et al., 2020). *Abbreviations*: AA, ascorbic acid; A_1_ and A_2_, helper SSDNA; A_3_, secondary target DNA; ABEI, N-(4-Aminobutyl)-N-(elthylisoluminol); (A:C-MB:B), three stranded substrate complex; AF, Alexa fluor; [Ag(Bin)]_n_, silver based benzimidazole polymer; AuNP, gold nanoparticles; bioHP1, biotinylated hairpin probe 1; bioHP2, biotinylated hairpin probe 2; BP, black phosphorus; BSA/Fc/S2, bovine serum albumin labeled with ferrocene and DNA strand S2; CDNM, controlled 3D DNA nanomachine; CHA, Catalytic hairpin assembly; CP, capture probe; CS, chitosan; CTAB, hexyltrimethyl ammonium bromide; CtDNA, circulating tumor DNA; CTQDs, CdTe quantum dots; dep/Au, electrodeposited with gold particles; 3DNM, 3D DNA nanomachine; DM, DNAzyme; dNTPs, deoxyribose nucleotide triphosphate; DPA, 9,10-diphenylanthracene; DPV, differential pulse voltammetry; 3D-rGO, three dimensional reduced graphene oxide; DW, DNA walker; ECL, electrochemiluminescence; Fc, Ferrocene; (Fc-DNA-Fc), double labeled ferrocene quencher probes; F, fuel; GCE, glassy carbon electrode; GH2, graphene oxide with hairpin 2; GH3, graphene oxide with hairpin 3; G-quad, G-quadruplex structure; H, hairpin chain; Hemin-G-quStr, Hemin/G-quadruplex structures; HDPC, chlorohexadecyl pyridine; HPdNs, hollow palladium nanospheres; HT, hexanethiol; LR, linear range; MBS, maleimidobenzoic acid N-hydroxy-succinimide ester; MCs, microcrystals; MCH, modified carbon hairpin chain; miR, microRNA; Mg^+2^, magnesium ion cofactor; MMB, magnetic micro beads; MT, mimic targets; NC1, Nanocomposite 1; NC2, Nanocomposite 2; NC3, nanocomposite3; NFs, nanoflowers; NPs. Nanoparticles; NS, nanosheet; P, protected strand; Pe, perylene; PFO, poly (9,9-di-n-octylflurenyl-2,7-diyl); Phi29, DNA polymerase; PP, protect probe; PSC, polystyrene microspheres; PtNCs, polyethyleneamine platinum nanoclusters; PVP, polyvinyl pyrrolidone; QDS, quantum dots; RP, reference probe; Ru (dcbpy)^2/3+^, tris (4, 4′-dicarboxyylic acid - 2,2′-bipyridyl) ruthenium II; S1, single strand; SA, streptavidin; SH, thiol modified hairpin; SP, signal probe; ss DNA, single strand DNA; ST, selected target; SWV, square wave voltammetry.

The ongoing progress in ultrasensitive biosensor development encompasses a wide array of approaches, introducing innovative techniques like DNA, tetrahedron, DNA walkers, ratiometric electrochemiluminescence (ECL) methods, and the integration of various nanoparticles and their modifications. With advancements in sensitivity and specificity in recent years, the boundaries of biosensing capabilities have been pushed by making it possible to detect biomolecules at extremely low concentrations with unprecedented precision. In this article, we have curated a selection of research findings that have demonstrated a linear range spanning from 0.01 to 1 × 10^8^ fM, accompanied by corresponding LOD ranging from 0.002 to 5 fM (Table 1). Additionally, we have specifically highlighted recent attempts aimed at developing ultrasensitive biosensors, achieving LOD below 0.009 fM (Figure 2).

Notable advancements include the work of Yang et al. (2021a), who introduced a groundbreaking ultrasensitive biosensor based on Ag NPs/SnO_2_ QDs/MnO_2_ nanoflowers (NFs). Their innovation involved integrating three co-reaction accelerators to expedite charge transfer, ultimately revealing catalytic active sites. The resultant “on-off-super on” ECL biosensor was coupled with a 3D DNA walker, enabling the remarkably sensitive detection of miR-21 (0.002 fM) (Yang et al., 2021a) (Figure 2D). Another groundbreaking approach was proposed by Wang et al. (2020a) involving the creation of a tripedal DNA walker through DNA self-assembly. This walker, which employed a catalytic hairpin assembly (CHA) method, moved along a track strand-functionalized electrode and facilitated ultrasensitive ECL biosensing of miRNA (Wang et al., 2020a). The DNA walker’s unique design demonstrated high efficiency in driving the detection process (Figure 2G).

Liao et al. (2020) introduced an ingenious approach utilizing 9,10-diphenyl anthracene doped perylene microcrystals (DPA@Pe MCs) to mitigate aggregation-caused quenching (ACQ). This method harnessed spatial configuration alterations to enhance ECL response, resulting in an effective avoidance of ACQ-induced limitations (Liao et al., 2020) (Figure 2J). Qin et al. (2023) engineered an enzyme-free electrochemical sensor employing Ag@N,O-C hierarchical nanomaterials and an entropy-driven DNA walker. This construct enhanced active sites for DNA walking substrates, facilitating electron transmission, and enabling the ultrasensitive quantification of PIK3CA E545K ctDNA (Qin et al., 2023) (Figure 2B). Gao et al. (2022) designed an electrochemical sensor by merging 3D DNA NM with a TSDR, exhibiting robustness against environmental fluctuations leveraging their properties for sensitive miR-182 detection (Gao et al., 2022) (Figure 2C). In another approach (Zhu et al., 2019), DNA tetrahedron nanostructure was used based on dual amplified ratiometric biosensor with hybridization chain reaction (HCR) for the ultrasensitive detection of microRNA-133a (Zhu et al., 2019).

Liu et al. (2021) devised an innovative method involving the opposing effects of H_2_O_2_ on two distinct ECL emissions. This potential-regulated ECL ratiometric method leveraged glucose oxidase (GOx) in conjunction with HCR and strand displacement amplification (SDA) for ultrasensitive miR-155 detection (Liu et al., 2021) (Figure 2I). Zhao et al. (2021) innovatively employed black phosphorus (BP) NSs to modulate the emission of quantum CdTe QDs, leading to simultaneous cathodic and anodic ECL signals. Their approach, utilizing BP-CdTe QDs, H_2_O_2_ and tripropylamine as the cathodic and anodic co-reactants, respectively enabled an ultrasensitive miR-126 detection (Zhao et al., 2021) (Figure 2F). Wang et al. (2021a) developed ABEI@AuPd NPs loaded with ABEI and synthesized 3D-rGO@Au NPs to establish strong electron transmission channels. This setup significantly amplified the ECL signal, allowing the detection of miR-141 at a low concentration of 0.0319 fM (Wang et al., 2021a) (Figure 2E). Another investigation (Wang et al., 2020b) demonstrated that the integration of 3D DNM with PtNCs@Ru (dcbpy)^2/3+^ improved the efficiency and sensitivity of the ECL biosensor (Figure 2H). This enhancement can be attributed to the presence of multiple energy donor/acceptor pairs, the utilization of Pb^+2^ dependent DNAzyme-assisted target recycling amplification technology, and the incorporation of multiple ECL resonance energy transfer (RET) mechanisms. These features collectively resulted in a more efficient electron-transfer process, reduced energy loss, and ultimately, heightened RET efficiency (Wang et al., 2020b; Tian et al., 2023) (Figure 2H).

Li et al. (2023) explored the effect of core diameter and DNAzyme cantilever length on 3D DNA nanomachine (CDNM) efficiency. By optimizing these parameters, they enhanced the walking rate and activity space of the CDNM, leading to the ultrasensitive detection of miR-21 at 0.0331 fM compared to traditional DNMs (Figure 2A). Another study (Liu et al., 2012) reported an exceptional sensitivity of 0.002 fM by integration of target-assisted isothermal exponential amplification, combined with the utilization of fluorescent DNA-scaffolded AgNCs. The successful implementation of this method was exemplified by its application in detecting miRNA within real samples that include human pancreatic cancer (AsPc-1), prostate carcinoma (22Rv1), hepatocellular carcinoma (BEL-7404), cervical cancer cell lines (HeLa), and breast cancer (MDA-MB231) cell line for early diagnosis, thereby showcasing its feasibility, simplicity, and cost-effectiveness. By achieving such remarkable sensitivity levels, this method opened up new avenues for the quantitative, accurate, and reliable assessment of miRNA expression. Recent research (Molla and Youk, 2023) employed the use of carbon-based nanomaterials for the detection of both small molecules and biomolecules (Table 1). The authors compared the performance of different analytes by increasing sensitivity or selectivity via modifications to the electrode and catalytic system. Using different electrodes, sensing applications for CdS (cadmium sulfide) are addressed and categorized depending on their composition. For electroanalytic applications, many electrochemical techniques have been taken into consideration, including electrochemical impedance spectroscopy (EIS), cyclic voltammetry (CV), differential pulse voltammetry (DPV), and ECL.

In other attempts to enhance the performance of biosensors for miRNA detection, two distinct research teams achieved an astonishing level of sensitivity, successfully detecting miRNA at concentrations lower than 0.009 fM (Pang et al., 2016; Zhang et al., 2019a). Both studies adopted distinct strategies, underscoring the diversity in their approaches to reach the exceptional LOD (Pang et al., 2016; Zhang et al., 2019a). Pang et al. (2016) developed an electrochemical sensor by employing a PEC aptasensor configuration. The approach hinged on the utilization of ZnO-NSs combined with CH_3_NH_3_PbI_3_ QDs. The establishment of a heterojunction between CH_3_NH_3_PbI_3_ QDs and ZnO-NSs facilitated a notable increase in the PEC signal. This aptasensor architecture facilitated the precise and accurate identification of miR-155 at a level of 0.005 fM (Pang et al., 2016). While Zhang and co-researchers (Zhang et al., 2019b) introduced an ultrasensitive, label-free electrochemical biosensor leveraging palladium nanoparticles (PdNPs) alongside rolling circle amplification (RCA). The biosensor was constructed by affixing electrode-immobilized dual-functionalized hairpin probes, which successfully detected miRNA at a detection threshold of 0.0086 fM. This newly developed biosensor also showcased remarkable selectivity, repeatability, and stability (Zhang et al., 2019b). The hairpin probe sensors hold tremendous potential for advancing the realm of ultralow-level miRNA diagnostics, boasting extraordinary levels of selectivity, repeatability, and stability (Meng et al., 2020).

Comparatively, Xu et al. (2020) reported the construction of a biosensor that relied on a novel DNA circular capture probe equipped with multiple target recognition domains achieving an LOD of <0.05 fM. They employed a mimetic proximity ligation assay which facilitated the capture of beacons labeled with ferrocene (Fc)-A1 and methylene blue (MB)-A2 to detect miRNAs (Xu et al., 2020). By comparing this approach with the conventional strategies of electrochemical biosensing using label-free (Cui et al., 2019) or label-based configurations with different electrochemical techniques such as amperometry, DPV, SWV, EIS, and potentiometry, they not only increased the reaction concentration but also avoided interference from capture probes (Munusami et al., 2022).

In another unique approach, Lu et al. (2020) developed an electrochemical sensor by combining 3D DNM with a TSDR. The signal of Fc-labeled dsDNA was reversely proportional to target miR-182 while the signal of AuNP-SA MMBs remained stable. The method offered a strong ability to eliminate interference from environmental changes, thus the enlarged AuNP-SA MMB depicted a detection limit of 0.058 fM (Lu et al., 2020). In contrast, when a similar approach was used by replacing SA with Fe_2_O_3_ the LOD decreased to 0.12 fM (Gao et al., 2022) (Figure 2C). Therefore, this significant advancement resulted in greatly improved detection sensitivity by declining hindrance from intricate biosystems (Lao et al., 2006; Várallyay et al., 2008).

Reported enhancements to sensor performance have been achieved through modifications involving various ligands, encompassing thionine (Deng et al., 2018), Pd (Gao and Yu, 2007b; Wang et al., 2021a), SA (Hao et al., 2017), silver sulfide (Ag_2_S) (Miao et al., 2016), iron oxides (Fe_2_O_3_/Fe_3_O_4_) (Yu et al., 2017; Zhou et al., 2017), cerium oxide (CeO2) (Deng et al., 2018; Liu et al., 2018; Chen et al., 2022), titanium dioxide (TiO2), GO (Erdem et al., 2017; Bao et al., 2019), MOFs (Liang et al., 2019; Sun et al., 2020), PAn (Peng et al., 2010; Wang et al., 2015b) and MXenes (Mohammadniaei et al., 2020). The enhancement of both LOD and miniaturization can be amplified by synergistically amalgamating diverse nanoparticle compositions and modification strategies that encompass ligands. The employment of appropriate ligands not only underscores the adaptability of nanoparticles in sensor design but also showcases the potential for attaining elevated sensor performance across a multitude of applications (Szunerits et al., 2022).

### Electrochemical sensing methods

Electrochemical techniques have garnered significant attention in the field of biosensing due to their numerous advantages including high sensitivity, cost-effectiveness, rapid analysis, low detection limits, user-friendly operation, and portability. Figure 1 illustrates a range of widely employed electrochemical sensing methods in biosensors. This comprehensive suite of electrochemical techniques empowers biosensing with a diverse array of tools to cater to various analytical requirements. It's important to highlight that the wide range of LOD values seen in the studies listed in Table 1 underscores the critical role of careful selection of sensing methods and nanocomposites, as both can significantly impact the final sensor performance.

Voltammetry stands out as one of the most frequently employed detection methods, driven by its fundamental exploration of redox reactions, electron transfer at electrode surfaces, reaction kinetics, and reaction mechanisms. Diverse subtypes of voltammetry, including CV, DPV, and square wave voltammetry (SWV), offer distinct approaches to analysis. CV involves a potential variation over a constant time, while DPV employs potential pulses at specific time intervals (Elgrishi et al., 2018; Sabahat et al., 2023). Table 1 shows a range of detection limits associated with voltammetry sensing techniques, spanning from 67 to 0.0089 fM. The diversity in detection limits underscores the significance of considering the compatibility of techniques with the choice of nanomaterial and fabrication strategy.

Noteworthy achievements in detection sensitivity have been reported by Zhang et al. (2019b) and Xu et al. (2020) (Zhang et al., 2019b) who utilized DPV in conjunction with metallic nanoparticle-based biosensors. In a different study, Kangkamano et al. (2018) demonstrated miRNA-based biosensors employing CV and EIS, utilizing a modified electrode incorporating pyrrolidinyl peptide nucleic acid (acpcPNA), polypyrrole (PPy), and silver nanofoam (AgNF). The fabrication of AgNF was characterized through EIS while CV measured the resulting current (Kangkamano et al., 2018). This electrode modification aimed to heighten sensitivity and selectivity for the mRNA probe, achieving increased surface area and safeguarding against unwanted materials. CV, beyond its sensing applications, serves to characterize electrochemical processes transpiring on electrode surfaces. Notably, this electrochemical approach achieved an ultrasensitive biosensor with LOD of 0.20 fM (Kangkamano et al., 2018).

Within the realm of biosensing, PEC has captured the attention of researchers due to its unique capabilities. PEC sensing involves exciting photoactive material using light to generate charge species, such as electrons and holes. The transfer of these charge carriers plays a pivotal role in redox reactions and charge recombination dynamics. The photoactive material not only offers active sites to enhance reaction kinetics but also minimizes charge recombination events (Gao et al., 2020). Zhou et al. (2023), utilizing a Cu_2_O(PTB7-Th/PDA+) designed a PEC biosensor for miRNA detection. In this setup, (Poly ([2,6′-4,8-di (5-ethylhexylthienyl)benzo [1,2- b; 3,3- b] dithiophene] {3-fluoro-2 [ (2-ethylhexyl)carbonyl]thieno [3,4-b]thiophenediyl}) (PTB7-Th) boosts Cu_2_O signals, facilitating charge separation in the bulk material, while *N*,*N*-bis(2-(trimethyl ammonium iodide)propylene)perylene-3,4,9,10 tetra -carboxydiimide (PDA^+^) acts as a mediator for charge transfer. To amplify signals, a 3D DNA walker connected to a dumbbell HCR was employed. The photoanode electrode, when exposed to light, exhibited substantially increased peak current compared to the pristine material, attributable to enhanced electron and hole movement and separation (Zhou et al., 2023). Another research group (Cui et al., 2023) employed a similar PEC sensing method for methylated RNA protein detection, utilizing a molybdenum diselenide/bismuth oxide (MoSe_2_/BiO) heterojunction as the photocathode. This heterojunction was synthesized via an *in-situ* method to augment MoSe_2_ activity. Signal amplification was achieved using poly aspartic acid-loaded alkaline phosphatase, resulting in an improved LOD (Cui et al., 2023). In a different study by Liu et al. (2020), a TI_3_C_2_/CdS nanocomposite was deposited on a Fluorine-doped Tin Oxide (FTO electrode), with chitosan as a binder for miRNA. Signal enhancement for PEC detection was accomplished using TMPyP, acting as an amplification agent, thereby enhancing sensitivity (Liu et al., 2020). These innovative approaches in PEC biosensing showcase the potential for highly sensitive and selective detection through synergistic interactions between photoactive materials, charge transfer mediators, and signal amplification strategies.

The electrochemical impedance spectroscopy (EIS) serves as a widely utilized technique for investigating the rate of electron transfer and diffusion in electrochemical reactions (Table 1). Through impedance analysis, the interaction between the electrode and the surface can be effectively probed by modulating the current (Walcarius et al., 2013; Naresh and Lee, 2021). A recent advancement involves an electrochemical biosensor employing reduced graphene oxide (RGO) and AuNPs to detect miR-128, showcasing sensitivity with a LOD of 0.08761 fM and 0.00956 fM using label-free and labeling approaches, respectively (Mohammadnejad et al., 2023). Kim and Kang (2023) developed an electrochemical biosensor using a graphitic nano-onion/molybdenum disulfide (MoS_2_) NSs composite for the detection of human papillomavirus (HPV)-16 and HPV-18 which will help in early diagnosis of cervical cancer (Kim and Kang, 2023). The acyl bonds on the surfaces of functionalized nano-onions and the amine groups on functionalized MoS_2_-NSs were chemically combined to create the electrode surface for testing DNA chemisorption, inducing an alteration in the electrochemical signal. When used as a sensing technique, this novel biosensor achieves LOD of 0.00696 fM using DPV and produces a current signal along with background noise. EIS, on the other hand, helps assess the developed electrode (Kim and Kang, 2023). These developments underscore the vital role of EIS in refining biosensing capabilities, allowing for sensitive and specific detection with diverse applications, from AuNP-based sensors to targeted DNA-triggered diagnostic tools.

A field-effect transistor (FET) is a specific type of transistor that harnesses electric fields to facilitate the conduction of electrons between its three essential electrodes: the source, drain, and gate electrode. Its functionality pivots around the control of material conductivity, achieved by manipulating the electric field of the gate electrode relative to the other electrodes. Depending on the semiconductor’s dopant and structure, the potential applied to the gate electrode can lead to either electron absorption or elimination within the channel (Grieshaber et al., 2008; Thriveni and Ghosh, 2022). Consequently, this process enables the adjustment of the depletion region, thereby shaping and reshaping the channels. This orchestration governs the conductance between the source and drain electrodes. This FET framework proves apt for amplifying weak signals and accommodating high impedance in biosensors (Grieshaber et al., 2008).

By substituting the gate electrode with a bio-sensitive surface in contact with a supporting solution, the FET can seamlessly transform into a biosensor. This configuration enables the FET biosensor to detect subtle changes caused by interactions between the bio-sensitive surface and target analytes, allowing for sensitive and selective measurements (Wang et al., 2020a; Liu et al., 2020). Li et al. (2021) research focused on a FET built on a foundation of CNTs, aimed at detecting exosomal miRNA associated with breast cancer. This biosensor is composed of CNTs functioning as a floating gate, a thin yttrium oxide (Y_2_O_3_) layer acting as an insulator, and AuNPs serving as linkers for probe capture. The detection of the target probe is accomplished by monitoring changes in current, resulting in heightened sensitivity and an impressively low LOD (0.00087 fM) (Li et al., 2021).

ECL stands as a chemiluminescent process, distinguished by the emergence of a luminophore at the surface of an electrode through the application of an electric voltage. This electrical manipulation triggers the transfer of high-energy electrons, ultimately generating an excited state that gives rise to luminescent signals. In ECL systems, nanomaterials act as catalysts to amplify the activation of molecules. This catalytic activity leads to the formation of oxidizing and reducing agents, which subsequently engage with the luminophores, culminating in the creation of electronically excited molecules. Co-catalysts plays an important role in this process and profoundly influence the activation of molecules and the resulting ECL phenomena (Jiao et al., 2023). Wang et al. (2021a) developed an innovative biosensor using DNA walkers and AuPd nanomaterials to achieve highly sensitive (low 0.0319 fM) detection of miR-141, employing the ECL technique. The design of the biosensor employs graphene as a conducting layer, while the inclusion of AuPd nanoparticles serves as an accelerator, enhancing the ECL signals.

As shown in Table 1, an array of studies has used amperometry as their preferred detection method for a variety of miRNAs. The exceptional sensitivity of this technique is based on the precise measurement of current during the electroactive material’s redox reaction. As seen in the study by Cai et al. (2013) an exceptionally low LOD (<0.05 fM) was achieved through the utilization of a gold electrode. This specific technique is particularly geared towards detecting metal ions, with its efficacy stemming from the selective reduction of only metal ions (Cai et al., 2013). However, the diverse range of LOD values recorded through amperometry can be ascribed to various factors, including the distinct nanocomposites chosen, the methodologies implemented, and the selection of appropriate sensing techniques (Table 1).

In another study, Yang et al. (2021b), engineered an ultrasensitive biosensor tailored for the detection of HPV miRNA. Within this biosensor, authors deployed a triple signal amplification strategy that ingeniously combined AuNPs with reverse transcription loop-mediated isothermal amplification (RT-LAMP) and a high-affinity biotin-avidin system. This multi-pronged approach yielded an extraordinary LOD, reaching an impressive 0.08 fM. Notably, this sensing paradigm incorporated the use of EIS for electrode fabrication assessment, while the performance stability, specificity, and miRNA detection were evaluated through amperometry. This adoption of the amperometric method bears notable advantages, primarily in the capability to discern HPV miRNA copies across a range spanning from 10^1^–10^8^ fM. The synergy of EIS and amperometry contributes to the overall efficacy of the biosensor, ensuring robust performance and the ability to detect analytes. The intricate combination of these techniques underscores the biosensor’s exceptional sensitivity and its potential to revolutionize the field of biosensors (Yang et al., 2021b).

Zhang et al. (2019a) engineered a biosensor centered on a multi-step process. This novel biosensing approach utilized duplex-specific nuclease (DSN)-assisted target recycling, followed by the integration of AuNPs and enzymatic signal amplification, all aimed at the precise detection of miR-21. Notably, the amperometric method emerges as a key player in signal amplification, operating on the principle of monitoring current changes over time. To ascertain the feasibility of this biosensor, CV was judiciously employed. CV enables the identification of miRNA presence through the observation of peak elevation (Zhang et al., 2019a).

Li et al. (2023) utilized a CDNM-based electrochemical biosensor to detect miR-21 achieving an LOD of 0.0331 fM. In contrast to previous methods using various electrochemical detection techniques, a significant improvement in LOD was observed. For instance, fluorescence yielded an LOD of 0.01 fM, ECL ranged from 2.44 to 4.92 fM, PEC exhibited 0.29 fM, and electrochemical methods ranged from 0.27 to 2.20 fM. The CDNM-based biosensor showcased a broader response range and lower sensing limit for miRNA detection due to the synergistic amplification from the combination of CDNM and TSDRs (Li et al., 2023).

Furthermore, the biosensor’s fabrication underwent rigorous investigation through EIS ensuring the optimal construction of the sensor, with LOD of 0.0433 fM. This achievement highlights the biosensor’s unparalleled sensitivity, rooted in the orchestrated amalgamation of DSN-assisted target recycling, AuNPs, and enzymatic signal amplification. The strategic integration of amperometric signal amplification, combined with the use of CV and EIS, demonstrates the advanced nature of this biosensing platform, ultimately leading to exceptionally accurate and reliable detection of miR-21 (Zhang et al., 2019b).

In summary, the choice of sensing technique depends on the unique characteristics of the material under investigation and construction strategies. Several factors drive the suitability of modified electrodes in this context. One prominent rationale stem from the fact that certain materials fail to manifest a discernible response within the potential range of conventional solid electrodes. To overcome this limitation, a higher potential is often necessitated, leading to the generation of a more pronounced background current. This scenario can consequently lead to a reduced LOD, impacting the sensor’s sensitivity. Furthermore, the employment of modified electrodes is warranted due to the potential for surface deactivation. The adsorption of biological molecules onto the electrode surface can significantly impact its stability, potentially compromising the accuracy and reliability of measurements. This is a crucial consideration, as the stability of the electrode is paramount for maintaining consistent and reproducible results.

Most biosensors employed in biomedical applications require a sizeable sample to detect an object, which may lead to false-positive or false-negative results. Only a few biosensors have been successful in the marketplace globally. More research is required in this area, and we anticipate that businesses will soon transform the scholarly work now being done into commercially viable prototypes.

The development of ultra-sensitive biosensors is still faced with several formidable challenges, each influencing the ultimate detection capabilities. One of the primary concerns is the need to achieve high specificity in biosensors. Complex samples often contain interfering compounds, which can lead to erroneous results, including false positives or false negatives. Gown (2016) conducted a study that highlighted false-negative results caused by various factors such as inadequate sensitivity, poor sample preparation, insufficient calibration, or interference from other substances. Additionally, the cost associated with the production and development of biosensors has limited their widespread use in various applications. The expenses can be significant, encompassing manufacturing, calibration, and integration costs related to immobilizing, purifying, and storing components. On the other hand, legal and ethical challenges also exist, including issues related to safety, quality, validation, standardization, and approval, which can vary from country to country and market to market (Sharma et al., 2015; Fogel and Limson, 2016).

Reproducibility is a critical aspect of biosensor development, influenced by multiple variables, including the quality of materials used, intricacies in the production process, and prevailing environmental conditions. The condition of electrode surfaces and unintended substance adsorption can significantly impact the challenges faced in biosensor development, making replication and electrode regeneration difficult tasks (Carpenter et al., 2018; Naresh and Lee, 2021). Naresh and Lee (2021) proposed several approaches for the replication of biosensors, including inkjet printing, screen printing, and microcontact printing. Further research is warranted to enhance the efficiency and effectiveness of these techniques.

Analyte detection in biosensors presents additional challenges, such as potential loss, diffusion, non-specific binding, and analyte degradation during their delivery to the electrode surface, as observed in various studies (Varshney and Mallikarjunan, 2009; Elgrishi et al., 2018; Vu and Chen, 2019; Naresh and Lee, 2021; Manimekala et al., 2022; Thriveni and Ghosh, 2022). Recent efforts have focused on addressing these obstacles and devising innovative strategies to enhance biosensor performance and reliability. In the context of FET biosensors, maintaining robust electrical performance and stability in liquid environments remains a significant concern (Vu and Chen, 2019; Cho et al., 2020; Manimekala et al., 2022). Vu and Chen (2019) enhanced the anti-interference capabilities of FET biosensors by addressing the issue of non-specific binding between unmodified linkers and targets through the mitigation of blocking surface sensors.

Recent advances in the realm of ultra-sensitive biosensors have been witnessed across various categories, including optical biosensors, colorimetric biosensors, nano-electronic biosensors, MOFs-based biosensors, and aptamer-based sensors. Despite these commendable developments, a notable observation is the disproportionate attention given to the calculation of LOD and linear range, while essential validation parameters, crucial for the establishment of an ultrasensitive electrochemical sensor, namely, precision, accuracy, repeatability, selectivity/specificity, linearity, and limit of quantification, have received relatively less emphasis and scrutiny.

## Conclusion

In conclusion, the landscape of biosensor development has seen a transformative shift towards achieving ultrasensitive detection capabilities. Innovative methodologies such as novel DNA walker strategies, controllable 3D DNM, advanced and ultrasensitive biosensing methods such as ratiometric ECL techniques, and nanoparticle modification with ligands integration have revolutionized the field, enabling the detection of biomolecules at unprecedentedly low concentrations with remarkable precision. These innovations have ushered in a new era in biosensing, empowering the precise detection of biomolecules at previously unimaginable low concentrations while ensuring exceptional precision and accuracy. The review has highlighted a selection of groundbreaking research findings, illustrating linear detection ranges spanning from 0.01 to 1 × 10^8^ fM and corresponding LOD ranging from 0.002 to 5 fM. Some studies have even surpassed these benchmarks, achieving LOD levels below 0.009 fM. The adaptability of nanoparticles in sensor design and the potential for elevated performance across various applications have been prominently demonstrated. Continued research in this domain is expected to yield further enhancements, opening up new horizons for applications in diagnostics, disease monitoring, and biomedical research. Collectively, these innovations mark significant progress in biosensing technologies, carrying profound implications for the field of diagnostic research.
